# Effects of vitamin D supplementation on vitamin D status and physical performance in football players: A systematic review and meta-analysis

**DOI:** 10.1371/journal.pone.0362552

**Published:** 2026-11-06

**Authors:** Jinfa Gu, Yu Zhang, Luhua Xiao

**Affiliations:** Qilu Medical University, Zibo, China; SPRINT - Sport Physical Activity and Health Research & Innovation Center, PORTUGAL

## Abstract

**Background/Objectives:**

Low vitamin D status is frequently reported among athletes, but whether vitamin D supplementation improves physical performance in football players remains uncertain. This systematic review and meta-analysis aimed to evaluate the effects of vitamin D supplementation on serum 25-hydroxyvitamin D [25(OH)D] concentration, jump performance, sprint performance, aerobic capacity, and muscular performance in association football and futsal players.

**Methods:**

PubMed, Embase, the Cochrane Library, Scopus, and Web of Science were searched from database inception to 31 May 2026. Controlled intervention studies comparing vitamin D supplementation with control conditions were eligible. Standardized mean differences were calculated as Hedges’ g and pooled using random-effects meta-analyses. Two reviewers independently extracted data and assessed risk of bias using RoB 2 or ROBINS-I. The certainty of evidence was assessed using GRADE. The protocol was prospectively registered in PROSPERO (CRD420261430873).

**Results:**

Eleven reports representing nine independent studies and 312 players were included. In RCT-only primary analyses, vitamin D supplementation was associated with higher circulating 25(OH)D concentrations (g = 1.41, 95% CI 0.90 to 1.92; I² = 58.0%). Pooled effects were not statistically significant for jump performance (g = 0.21, 95% CI −0.10 to 0.53), sprint performance (g = −0.03, 95% CI −0.40 to 0.34), aerobic capacity (g = 0.00, 95% CI −0.45 to 0.45), or muscular performance (g = 0.19, 95% CI −0.14 to 0.51). The certainty of evidence was low for circulating 25(OH)D, jump performance, and muscular performance, and very low for sprint performance and aerobic capacity.

**Conclusions:**

Vitamin D supplementation was associated with increased circulating 25(OH)D concentrations, whereas current evidence is insufficient to support a consistent improvement in physical performance. Given the low to very low certainty of evidence, these findings should be interpreted cautiously. Larger, well-designed randomized controlled trials using standardized, football-relevant performance tests are warranted.

## 1. Introduction

Association football players repeatedly perform short-distance sprints, jumps, accelerations, decelerations, and changes of direction. These activities depend on multiple physical capacities, including sprint and jump performance, aerobic capacity, and muscular strength and power [1]. Low vitamin D status is not uncommon among athletes. Using a serum 25-hydroxyvitamin D [25(OH)D] threshold of <50 nmol/L, prevalence rates of 30% among adult elite athletes and 39% among youth elite athletes have been reported [2].

Vitamin D status is usually determined by serum 25(OH)D levels [3]. The presence of vitamin D receptors in skeletal muscle provides a biological rationale for its potential involvement in the modulation of skeletal muscle performance and function [4,5]. However, this biological plausibility does not establish that vitamin D supplementation necessarily improves athletic performance. In one review of 13 randomized controlled trials (RCTs), seven studies assessed physical performance; although supplementation improved 25(OH)D status, none of these studies reported a statistically significant effect on physical performance [6].

Evidence also varies across specific performance domains. Previous meta-analyses have reported no significant improvement in overall muscular strength or power, although favorable findings have been observed in specific lower-limb or quadriceps strength outcomes [7,8]. Similarly, no statistically significant improvement was found in analyses restricted to maximal strength and vertical jump power [9].

Available studies and meta-analyses have involved heterogeneous athletic groups and performance tests, which might overlook effects that are only pertinent to some sports with different intermittent high-intensity requirements, like football [6,7,10–13].

Some reviews emphasized muscular strength and power; others provided narrative syntheses of a variety of performance outcomes [6,7]. These findings may not be directly applicable to football players, given the differences in the physical demands of sports, training background, and performance-testing methods. The sport-specific evidence-synthesis framework adopted in the present study was applied to football and futsal players. Serum 25(OH)D concentrations, jump performance, sprint performance, aerobic capacity, and muscular performance were separately synthesized quantitatively. This approach also distinguishes the serum 25(OH)D response from the effects of supplementation on athletic performance and assesses whether supplementation produces measurable functional changes.

Therefore, an evaluation and quantification of the effects of vitamin D supplementation on serum 25(OH)D concentrations, jump performance, sprint performance, aerobic capacity, and muscular performance in football players was the primary objective of this systematic review and meta-analysis. Exploratory subgroup analyses of circulating 25(OH)D examined baseline vitamin D status and supplementation regimen. To test the robustness of the conclusions, sensitivity analyses were performed, and the certainty of the evidence was subsequently assessed using the GRADE framework for each key outcome.

## 2. Materials and methods

### 2.1. Registration and reporting standards

To report this systematic review and meta-analysis, the PRISMA 2020 statement was consulted, and the methodological recommendations in the Cochrane Handbook for Systematic Reviews of Interventions guided the planning and execution of each phase of the review process [14]. Before data extraction and analysis began, the study protocol was prospectively registered in the International Prospective Register of Systematic Reviews (PROSPERO; registration number CRD420261430873).

Ethics approval was not applicable, as this study was based exclusively on data from previously published studies.

### 2.2. Search strategy and study selection

Independent systematic searching was performed by the two reviewers in PubMed, Web of Science, Scopus, Embase, and Cochrane Library from inception to 31 May 2026. The keywords used in the search included controlled vocabulary terms (such as MeSH or Emtree, if available) and free-text keywords related to vitamin D supplementation and football players. The terms used in searching the core concepts were football, soccer, futsal, vitamin D, vitamin D3, cholecalciferol, and 25-hydroxyvitamin D, as well as their synonyms and spelling variations. Appropriate Boolean operators were used as needed. For the search, no restrictions were made on sex, age, competitive level, or year of publication; these were assessed in the screening process. Relevant reviews and reference lists of included studies underwent manual searching as well.

After completing the search process, the researchers imported all located records into a reference management program, and they were de-duplicated before being independently screened by two reviewers. Screening was initially done at the title-abstract level, and then at the full-text level based on criteria for eligibility. Disagreements between reviewers were resolved through discussion or, when necessary, consultation with a third reviewer. Search strategies are provided in detail in Supplementary S1 Table.

### 2.3. Eligibility criteria

The eligibility criteria were defined a priori according to the population, intervention, comparator, outcomes, and study design (PICOS) framework. Association football and futsal players were eligible for this study. Meanwhile, rugby or mixed-sport studies were excluded.

Interventions that were eligible were oral vitamin D supplementation and supplementation by any other clearly specified route, and no minimum or maximum dosage or length of intervention was specified. Articles were excluded if they focused on multiple nutritional interventions. Placebos, no supplementation, usual training, or a non-vitamin D control condition were all eligible comparators.

Investigations were deemed eligible for inclusion if they presented at least one of the subsequent outcomes: 25(OH)D concentration, jump performance, sprint performance, aerobic capacity, or muscular performance. RCTs, placebo-controlled trials, non-RCT studies, and quasi-experimental controlled studies were all considered eligible designs. Case reports, reviews, conference abstracts, non-peer-reviewed articles, and studies without extractable data or with a single-arm pre-post design were excluded. Only those articles that were available as full text and published in the English language were deemed eligible.

### 2.4. Data extraction and data handling

A pre-designed standardized form was used to extract data independently by two reviewers. The information collected included the demographics of the subjects, intervention length, study design, comparator intervention, sample size, vitamin D intervention, baseline 25(OH)D levels, and year of publication. Data were extracted from published reports, tables, figures, and supplementary materials as appropriate.

If there were duplicate or overlapping reports, the data for each report was not counted more than once in any particular pooled analysis. Results were classified according to the analytical framework used in this review. Serum 25(OH)D concentration was used as the measure of vitamin D status. Physical performance outcomes comprised jump performance, sprint performance, aerobic capacity as measured by VO_2_max when available, and muscular performance as both strength and power measures. To prevent double-counting, one measure per outcome domain was selected for each independent comparison using a consistent hierarchy. Countermovement or vertical jump measures were prioritized for jump performance; the 10-m sprint was preferred for sprint performance, with the 30-m sprint used when 10-m data were unavailable; and VO_2_max was prioritized for aerobic capacity. For muscular performance, one lower-limb strength or power measure was selected without reusing a measure already included in the jump-performance domain. Subgroup data for 25(OH)D concentration were extracted for baseline vitamin D status and supplementation regimen.

Disagreements during data extraction were resolved through discussion and, if needed, consultation with a third reviewer.

### 2.5. Risk of bias and certainty of evidence

Risk of bias was evaluated for the specific outcome/result contributing to each quantitative synthesis. RCT results were assessed using the Cochrane RoB 2 instrument [15], whereas non-randomized and quasi-experimental results were assessed using ROBINS-I [16]. Outcome-specific judgments are reported in Supplementary S4 Table, with an overall graphical summary shown in Fig 2.

The GRADE framework was used to assess the certainty of evidence for each RCT-only primary outcome [17]. Certainty was rated as high, moderate, low, or very low according to risk of bias, inconsistency, indirectness, imprecision, and publication bias. Non-randomized and quasi-experimental evidence was included only in sensitivity analyses and did not contribute to the GRADE ratings. Two reviewers independently made these assessments, with disagreements resolved through discussion or consultation with a third reviewer.

### 2.6. Statistical analysis

Randomized comparisons were used for the primary meta-analyses and GRADE assessments, whereas eligible non-randomized and quasi-experimental comparisons were included only in sensitivity analyses. For all meta-analyses, random-effects models were applied, as the studies were likely to be clinically and methodologically heterogeneous [18]. To account for the variation in outcome reporting across studies, standardized mean differences (SMDs) with 95% CIs were calculated for continuous outcomes, and Hedges’ g was used to adjust for small-sample bias [19]. For circulating 25(OH)D, an additional absolute-scale analysis using mean differences was performed after harmonizing values to ng/mL.

In cases where the standard deviations of change scores were not provided, they were calculated using the formula below in conjunction with baseline and post-intervention standard deviations of the scores:


$$\begin{array}{c} {SD}_{\text{change}}=\sqrt{{{SD}^{\text{2}}}_{\text{baseline}}+{{SD}^{\text{2}}}_{\text{post}}-\text{2r}\times {SD}_{\text{baseline}}\times {SD}_{\text{post}}} \end{array}$$

When the pre–post correlation was not reported, a correlation coefficient of r = 0.5 was assumed for estimating change-score standard deviations. All pooled effect estimates were based on between-group differences in change scores; post-intervention values were not used as standalone meta-analytic inputs. Effect directions were harmonized before pooling so that positive values indicated effects favoring vitamin D supplementation. For sprint-time outcomes, effect sizes were reverse-coded because lower values indicate better performance.

Between-study variance (τ^2^) was estimated using restricted maximum likelihood (REML), and conventional Wald-type 95% CIs were used for the pooled random-effects estimates. No Hartung–Knapp adjustment was applied to the primary pooled-effect CIs. Statistical heterogeneity was summarized using τ^2^ and I^2^ [20]. The Q-profile method was used to determine the 95% confidence interval for τ^2^, and 95% prediction intervals were calculated for each main meta-analysis [21]. Exploratory subgroup analyses of 25(OH)D concentration were conducted by baseline vitamin D status and supplementation regimen. For the baseline-status subgroup analysis, vitamin D deficiency was operationally defined as baseline 25(OH)D < 20 ng/mL and insufficiency as 20–30 ng/mL. Between-subgroup tests were interpreted cautiously because of the small number of comparisons.

Sensitivity analyses included a leave-one-out analysis for the RCT-only 25(OH)D meta-analysis, comparison of random-effects and fixed-effect models, inclusion of eligible non-randomized and quasi-experimental comparisons, and alternative assumed pre–post correlations (r = 0.25 and 0.75) for change-score standard deviations that required reconstruction. Publication bias was planned to be assessed using funnel plots or regression-based tests only when at least 10 effect sizes were available for a given meta-analysis [22]. A two-sided threshold was used to determine statistical significance at p < 0.05. Statistical analyses were performed in R version 4.4.3 using the meta package.

## 3. Results

### 3.1 Study selection

Database searching revealed 659 records (Fig 1). 238 records were screened after the removal of 421 duplicates, with 211 excluded. Twenty-seven reports were sought for retrieval, and all were retrieved. 16 reports were excluded after full-text evaluation because of an ineligible population (n = 4), wrong intervention or study design (n = 5), no eligible outcome (n = 3), or no eligible comparator or control group (n = 4). In total, 11 reports, corresponding to 9 independent studies, were included in the meta-analysis.

**Fig 1:**
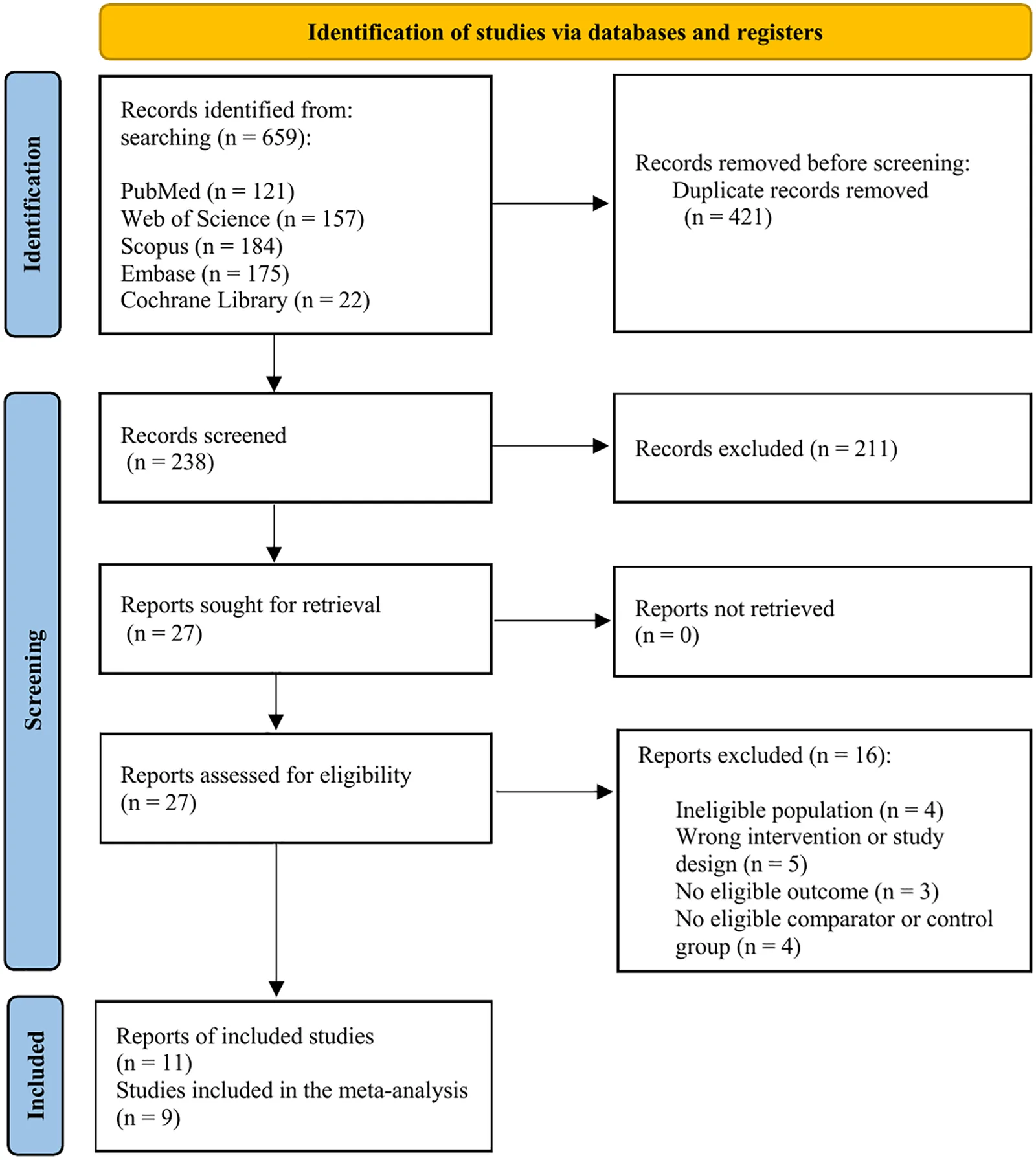
PRISMA flow diagram of the study selection process.

### 3.2. Study characteristics

Eleven reports, corresponding to nine independent studies, were included in this systematic review and meta-analysis [10,11,23–31]. After accounting for overlapping reports from the same participant cohorts, the included studies comprised 312 unique participants in total. Of the nine independent studies, seven were randomized controlled trials, whereas two were non-randomized or quasi-experimental controlled studies. Sample sizes ranged from 10 to 69. The included populations consisted of association football or futsal players, predominantly youth or young male athletes.

Intervention duration varied across studies, with 8-week interventions being the most common. Vitamin D supplementation protocols included daily or weekly oral supplementation, a single high-dose vitamin D3 bolus, and intramuscular vitamin D3 injection. Comparators included placebo, no supplementation, non-supplemented control conditions, or matched exercise-only conditions.

The outcomes extracted for synthesis were 25(OH)D concentration, jump performance, sprint performance, aerobic capacity, and muscular performance. A summary of the included studies’ characteristics is provided in Table 1.

**Table 1:** Characteristics of included studies.

Study	Participants / context	Design / comparison	Sample size	25(OH)D baseline / follow-up data, ng/mL	Vitamin D intervention	Duration / timing	Performance test(s) used	Outcomes extracted
Alimoradi et al., 2019	National football/futsal athletes; both sexes; young adults	Randomized controlled clinical trial; vitamin D vs placebo	N = 69 (35/34)	VitD 27.5 → 44.9; placebo 24.4 → 21.3	Oral; 50,000 IU/week × 8 weeks (planned total 400,000 IU)	8 weeks; season NR	Vertical jump; leg-press strength	25(OH)D; jump; muscular
Bezrati et al., 2020	Male recreational/amateur soccer players; 8–15 y; vitamin D insufficient	Randomized controlled trial; vitamin D vs placebo	N = 36 (19/17)	VitD 12.4 → 28.6; placebo 12.1 → 19.3	Oral single dose; vitamin D3 200,000 IU	12 weeks; winter	Vertical jump; 10 m sprint	25(OH)D; jump; sprint
Bezuglov et al., 2025	Male elite academy soccer players; mean age 13.0 ± 2.78 vs 12.3 ± 3.14 y; deficient	Randomized controlled trial; vitamin D vs no supplementation	N = 48 (25/23)	VitD 15.59 → 30.25; control 15.56 → 20.59	Oral; cholecalciferol 15,000 IU/week × 6 weeks (planned total 90,000 IU)	6 weeks	No performance outcome included in quantitative synthesis	25(OH)D
Brzeziański et al., 2022	Young male soccer players; 17.5 ± 0.70 y	Randomized placebo-controlled supplementation trial; vitamin D vs placebo/no vitamin D	N = 25 (12/13)	VitD 27.9 → 47.4; control 25.5 → 27.2	Oral; 20,000 IU twice/week × 8 weeks (planned total 320,000 IU)	8 weeks; winter preparation period (mid-Jan to mid-Mar)	CMJ; 10 m sprint; VO_2_max; 10-jump power	25(OH)D; jump; sprint; aerobic; muscular
Close et al., 2013	Male academy soccer players from an English professional club; analyzed subset n = 10; exact subset age NR	Randomized placebo-controlled supplementation trial	N = 10 (5/5)	VitD 11.6 → 41.2; placebo 21.2 → 29.6	Oral capsules; vitamin D3 5,000 IU/day × 8 weeks (planned total 280,000 IU)	8 weeks; UK winter	No performance outcome included in quantitative synthesis	25(OH)D
Jastrzębska et al., 2016; Jastrzębska et al., 2018; Skalska et al., 2019^a^	Well-trained youth soccer players; 17.5 ± 0.6 y; Polish top junior league; sex NR	Non-randomized controlled 8-week HIIT + supplementation trial; vitamin D vs placebo	N = 36 (20/16)	Baseline: VitD 19.4; placebo 19.0. Follow-up values NR; reported change +23.12 vs −1.60	Oral; vitamin D3 5,000 IU/day × 8 weeks (planned total 280,000 IU)	8 weeks; HIIT period	CMJ; 10 m sprint; VO_2_max; Wingate peak power	25(OH)D; jump; sprint; aerobic; muscular
Jastrzębska et al., 2022	Young soccer players; 17.2 ± 1.16 y; Polish Central Junior League; sex NR	Randomized placebo-controlled supplementation stage within a seasonal longitudinal study	N = 24 (11/13)	VitD 24.1 → 26.6; placebo 24.8 → 26.2	Oral; vitamin D3 5,000 IU/day for 8 weeks (planned total 280,000 IU)	Supplementation after T2; winter; T3 assessment in May 2020	CMJ; 10 m sprint; VO_2_max; 10-jump test	25(OH)D; jump; sprint; aerobic; muscular
Michalczyk et al., 2020	Professional soccer players; age/sex NR in report	Randomized placebo-controlled supplementation phase within a three-stage longitudinal study	N = 28 (15/13)	EG 32.37 → 54.03; placebo-group exact values NR	Oral; cholecalciferol 6,000 IU/day × 6 weeks (planned total 252,000 IU)	6 weeks; late Jan to Mar (winter)	30 m sprint; treadmill VO_2_max; leg power (PLL)	Sprint; aerobic; muscular
Motavari et al., 2022	Male futsal players with vitamin D deficiency; group mean ages 25.2–26.9 y	Four-arm quasi-experimental controlled study: EX, EXS, SUP, CON	N = 36 (9/group)	SUP 21.01 → 58.47; CON 18.96 → 19.22; EXS 19.92 → 56.53; EX 18.07 → 21.40	Intramuscular vitamin D3 50,000 IU every 2 weeks for 8 weeks; cumulative dose NR	8 weeks; participants selected in late summer	VO_2_max (Bruce test); lower-body strength	25(OH)D; aerobic; muscular

Notes. 25(OH)D values are shown as baseline→follow-up values for the vitamin D and comparator arms where directly reported. When a follow-up value was not directly reported, the reported baseline and change are shown instead. NR = not reported or not available for the analyzed comparison; CMJ = countermovement jump; PLL = power of the left leg; EG = experimental group; EX = exercise; EXS = exercise plus supplementation; SUP = supplementation; CON = control; HIIT = high-intensity interval training. Planned total vitamin D dose was calculated from the reported dose and duration only when the regimen was unambiguous. ^a^The Jastrzębska 2016/2018/Skalska 2019 reports were treated as one overlapping non-randomized trial to avoid double counting. Randomized comparisons contributed to the primary analyses; non-randomized/quasi-experimental comparisons were included only in sensitivity analyses.

### 3.3. Risk of bias

The outcome-specific risk-of-bias assessments are reported in Supplementary S4 Table, with an overall graphical summary shown in Fig 2. For the RCT results contributing to the primary analyses, overall outcome-specific judgments were some concerns for results from six trials and high risk for results from Jastrzębska et al. (2022); no included RCT result was rated as low risk overall. The main concerns involved the randomization process, deviations from intended interventions, missing outcome data, and selection of the reported result, whereas measurement of the included outcomes was judged as low risk. Outcome-specific results from the two non-randomized/quasi-experimental trials were judged as moderate risk overall under ROBINS-I, primarily because of concerns regarding confounding, selection of participants, deviations from intended interventions, and selection of the reported result.

**Fig 2:**
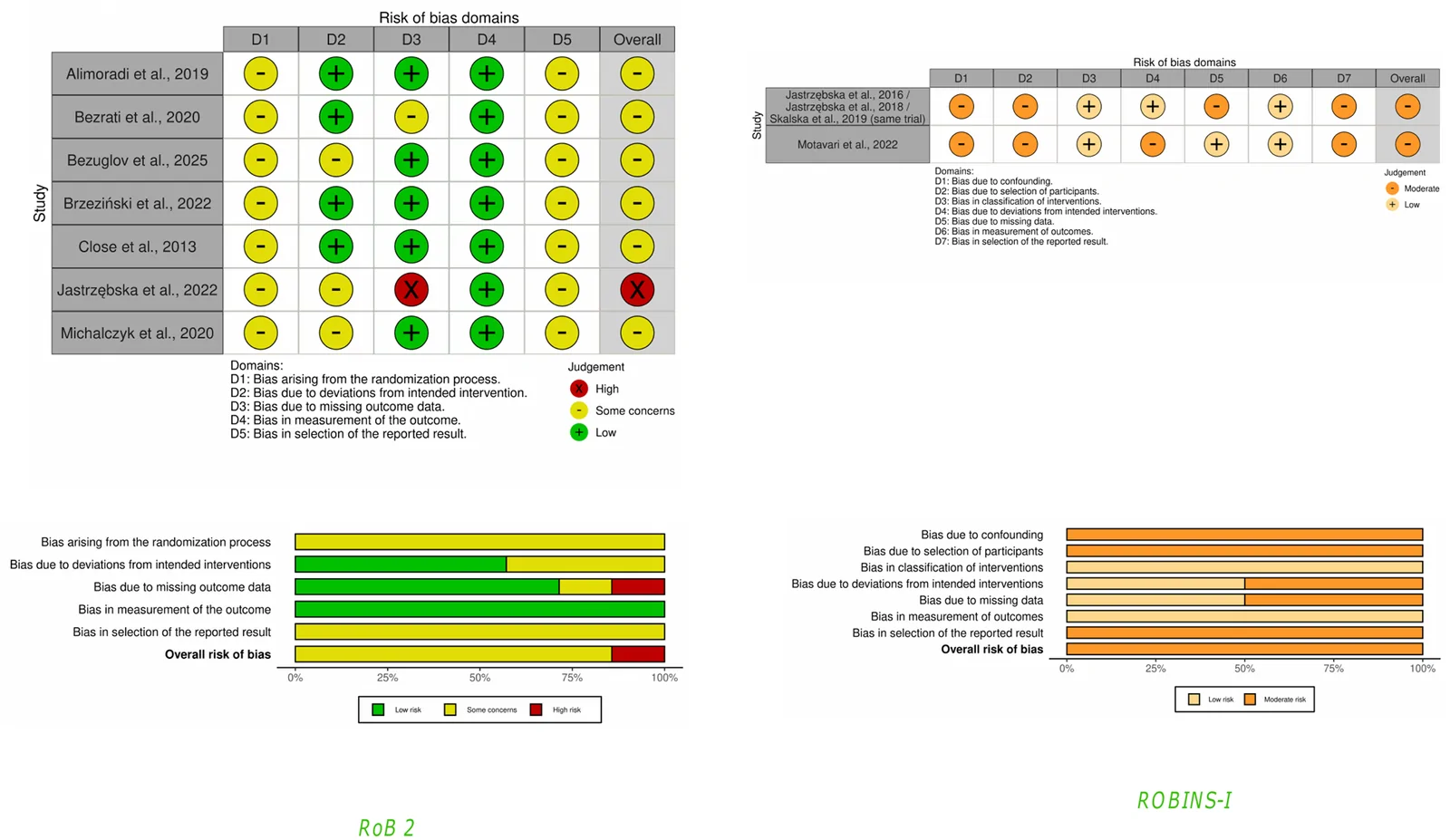
Summary of risk-of-bias assessments for included randomized and non-randomized studies. Detailed outcome-specific judgments are provided in Supplementary S4 Table.

### 3.4. Overall effects

In the RCT-only primary analyses, vitamin D supplementation was associated with a statistically significant increase in circulating 25(OH)D concentration compared with control conditions (k = 6, N = 212; Hedges’ g = 1.41, 95% CI 0.90 to 1.92; p < 0.001; I^2^ = 58.0%). An additional RCT-only absolute-scale analysis yielded a pooled mean difference of 12.14 ng/mL (95% CI 5.84 to 18.43; p < 0.001; I^2^ = 89.8%), with substantial heterogeneity remaining (S2 Table). No statistically significant pooled effects were observed for jump performance (k = 4, N = 154; Hedges’ g = 0.21, 95% CI −0.10 to 0.53; p = 0.186; I^2^ = 0%), sprint performance (k = 4, N = 113; Hedges’ g = −0.03, 95% CI −0.40 to 0.34; p = 0.886; I^2^ = 0%), aerobic capacity (VO_2_max) (k = 3, N = 77; Hedges’ g = 0.00, 95% CI −0.45 to 0.45; p = 0.995; I^2^ = 0%), or muscular performance (k = 4, N = 146; Hedges’ g = 0.19, 95% CI −0.14 to 0.51; p = 0.264; I^2^ = 0%). The corresponding RCT-only forest plots are shown in Fig 3, with detailed study-level forest plots and prediction intervals provided in Supplementary S1 Fig.

**Fig 3:**
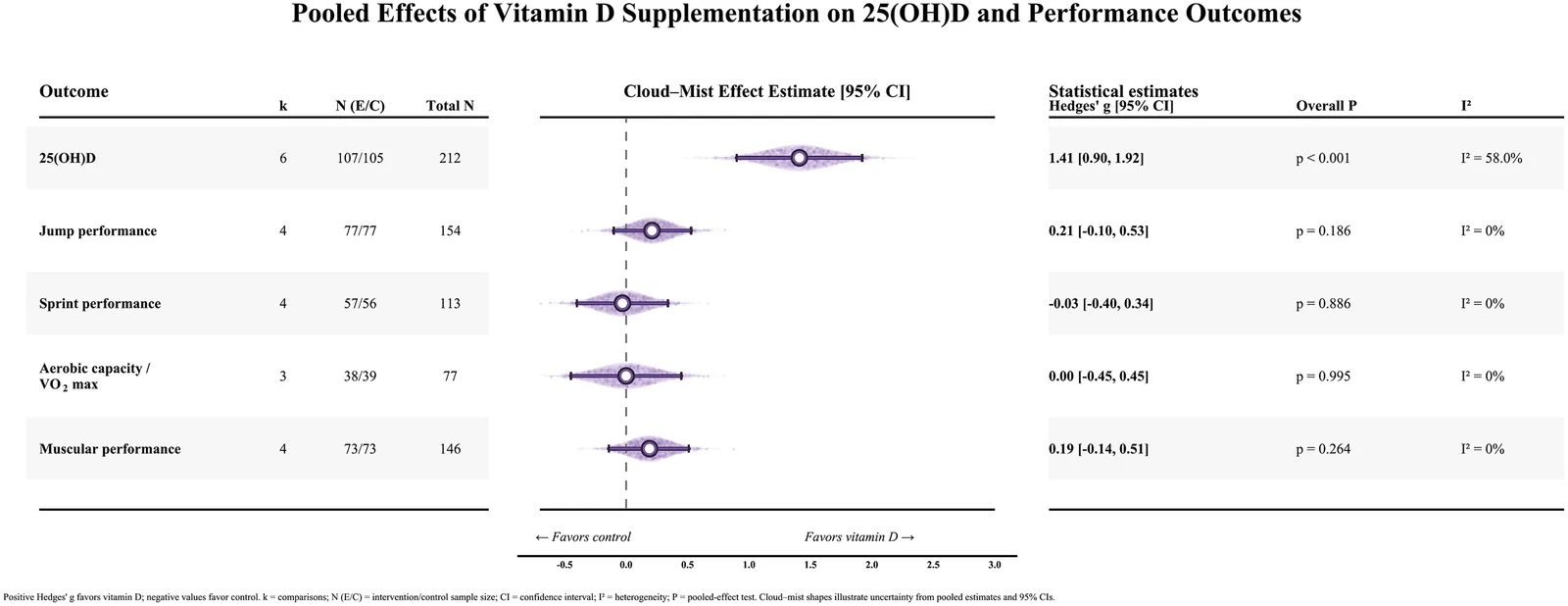
Forest plots of the effects of vitamin D supplementation on 25(OH)D concentration and performance outcomes. Positive values favor vitamin D supplementation.

### 3.5. Exploratory subgroup analyses of 25(OH)D concentration

Exploratory RCT-only subgroup analyses showed no statistically significant between-subgroup differences by baseline vitamin D status (p = 0.228) or supplementation regimen (p = 0.270) (Fig 4). Pooled Hedges’ g values were 1.76 (95% CI 1.25 to 2.26) and 1.10 (95% CI 0.25 to 1.94) for the deficient and insufficient subgroups, respectively, and 1.21 (95% CI 0.49 to 1.93) and 1.76 (95% CI 1.25 to 2.26) for the higher-dose/repeated oral and moderate/lower-dose or bolus oral regimen subgroups, respectively. Given the small number of comparisons, these analyses were considered exploratory and the between-subgroup tests were interpreted cautiously.

**Fig 4:**
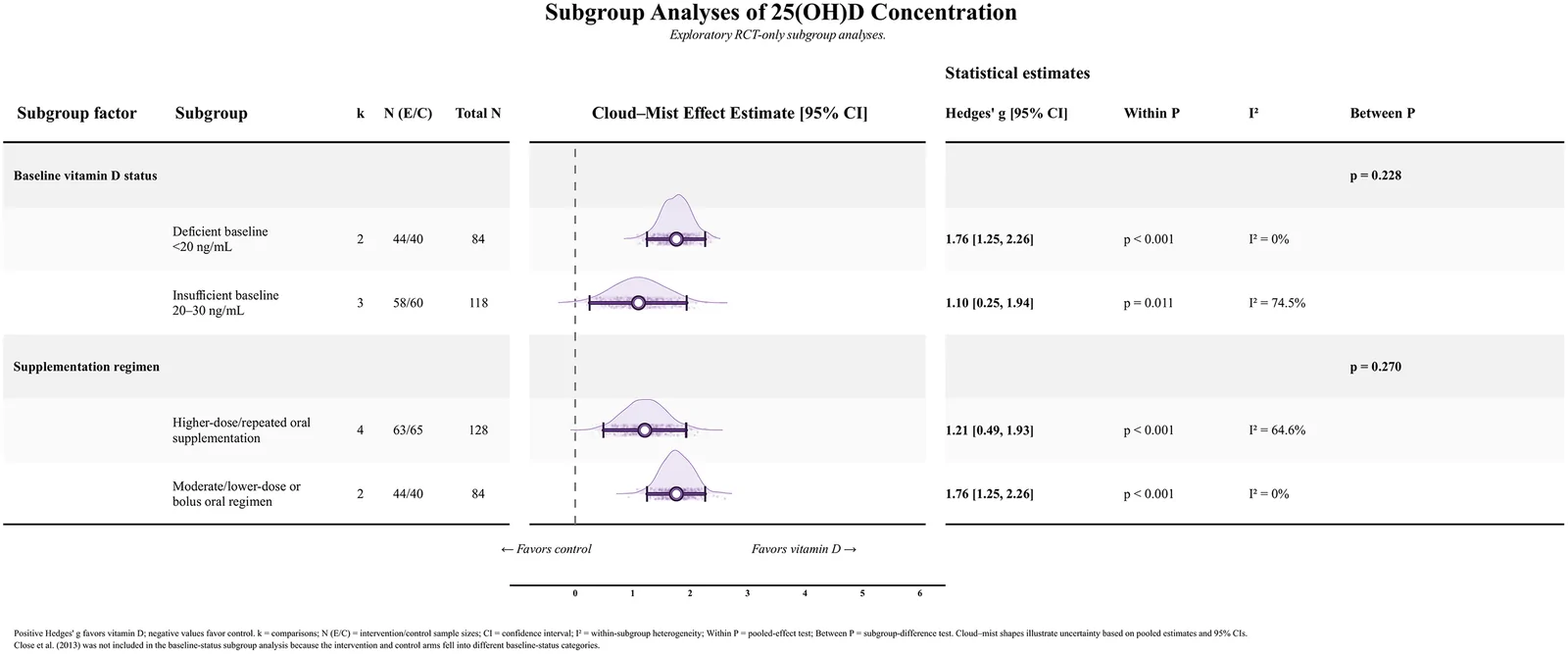
Exploratory RCT-only subgroup analyses of 25(OH)D concentration by baseline vitamin D status and supplementation regimen. Positive values favor vitamin D supplementation.

### 3.6. Sensitivity analyses

Sensitivity analyses including eligible non-randomized and quasi-experimental comparisons yielded the same overall interpretation: circulating 25(OH)D remained significantly increased, whereas pooled effects for all four physical-performance outcomes remained non-significant. The direction and statistical interpretation of the primary 25(OH)D finding were also consistent when a fixed-effect model was applied and when alternative pre-post correlation coefficients (r = 0.25 and 0.75) were used to reconstruct change-score standard deviations. Leave-one-out analyses of the RCT-only 25(OH)D meta-analysis yielded pooled Hedges’ g values ranging from 1.31 to 1.63, with all estimates remaining statistically significant (Supplementary S3 Table). Across the alternative correlation assumptions, pooled effects for jump performance, sprint performance, aerobic capacity, and muscular performance remained non-significant. Detailed results are provided in Supplementary S2 and S3 Tables.

### 3.7. Certainty of evidence (GRADE)

Table 2 provides an overview of the certainty of evidence for the RCT-only primary outcomes. Evidence was rated as low for 25(OH)D concentration, jump performance, and muscular performance, and very low for sprint performance and aerobic capacity (VO_2_max). Downgrading was mainly due to RoB across studies, substantial heterogeneity for 25(OH)D concentration, and imprecision for performance outcomes. No outcome was downgraded for indirectness. Because fewer than 10 effect sizes were available for each outcome, formal small-study-effect tests were not used. Publication bias was considered qualitatively within GRADE; no downgrade was applied, and this decision was not interpreted as evidence that publication bias was absent. The certainty of evidence for the pooled estimates was generally low to very low.

**Table 2:** Certainty of evidence (GRADE framework) for RCT-only primary outcomes.

Outcome	No. of participantsand effect sizes	Pooled estimate,Hedges’ g [95% CI]	Risk of bias	Inconsistency	Indirectness	Imprecision	Publication bias	Certainty of evidence(GRADE)
25(OH)D concentration	N = 212;k = 6	1.41 [0.90, 1.92]	Serious^a^	Serious^b^	Not serious	Not serious	Not downgraded^e^	⊕⊕◯◯Low
Jump performance	N = 154;k = 4	0.21 [−0.10, 0.53]	Serious^a^	Not serious	Not serious	Serious^c^	Not downgraded^e^	⊕⊕◯◯Low
Sprint performance	N = 113;k = 4	−0.03 [−0.40, 0.34]	Serious^a^	Not serious	Not serious	Very serious^d^	Not downgraded^e^	⊕◯◯◯Very low
Aerobic capacity (VO_2_max)	N = 77;k = 3	0.00 [−0.45, 0.45]	Serious^a^	Not serious	Not serious	Very serious^d^	Not downgraded^e^	⊕◯◯◯Very low
Muscular performance	N = 146;k = 4	0.19 [−0.14, 0.51]	Serious^a^	Not serious	Not serious	Serious^c^	Not downgraded^e^	⊕⊕◯◯Low

GRADE, Grading of Recommendations Assessment, Development and Evaluation; CI, confidence interval; N, total sample size; k, number of pooled effect sizes; VO_2_max, maximal oxygen uptake.

^a^Downgraded by one level for risk of bias because the contributing randomized-trial results were judged as having some concerns or high risk of bias in the outcome-specific RoB 2 assessment.

^b^Downgraded by one level for inconsistency because substantial statistical heterogeneity was observed for 25(OH)D concentration (I^2^ = 58%).

^c^Downgraded by one level for imprecision because the 95% confidence interval crossed the null effect and the total sample size was limited.

^d^Downgraded by two levels for imprecision because of the small total sample size and a wide 95% confidence interval crossing the null effect.

^e^ Because fewer than 10 effect sizes were available for each outcome, formal small-study-effect tests were not used. Publication bias was considered qualitatively within GRADE; no downgrade was applied, and this decision was not interpreted as evidence that publication bias was absent.

## 4. Discussion

### 4.1. Main Findings

This systematic review and meta-analysis included 11 reports from nine independent studies involving 312 football players. Vitamin D supplementation was associated with higher serum 25(OH)D levels compared with control conditions. However, the pooled effects on jump performance, sprint performance, aerobic capacity, and muscular performance were not statistically significant. The collected data must be interpreted cautiously in relation to the limited number of studies and participants and the low to very low certainty of evidence.

The present study targeted association football and futsal players only and separately combined jump performance, sprint performance, aerobic capacity, and muscular performance, while previous systematic reviews combined these outcomes in athletes of various sports. This framework mitigates concerns of indirectness of evidence obtained from several sports and does not combine different functional outcomes into a single overall performance measure.

### 4.2. Serum 25(OH)D response to supplementation

Consistent with findings reported in previous systematic reviews and meta-analyses involving athletic populations, vitamin D supplementation was associated with a measurable increase in serum 25(OH)D levels among football players [6]. Serum 25(OH)D is generally employed to examine vitamin D status, and the observed increase is consistent with the intended biochemical effect of supplementation [3]. The observed biochemical response is therefore consistent with an improvement in vitamin D status. However, it should not be interpreted as direct evidence of enhanced physical performance, because the pooled effects for performance outcomes were not statistically significant in the present review. The increase in serum 25(OH)D remained statistically significant in sensitivity analyses, although substantial heterogeneity remained across studies.

The absence of statistically significant pooled effects on performance outcomes despite improved vitamin D status may reflect the multifactorial nature of football performance [1]. The selection and sensitivity of performance tests may influence whether football-relevant adaptations can be detected, alongside training load, match exposure, individual fitness, nutrition, recovery, and other factors. Accordingly, the observed increase in serum 25(OH)D should not be assumed to translate into measurable improvements in sprinting, jumping, aerobic capacity, or strength. Such an interpretation is especially pertinent when players are evaluated on the results of their performances over a relatively short period of intervention, or for players who are physically well-trained.

Potential sources of heterogeneity in the 25(OH)D analysis include differences in baseline vitamin D status, supplementation dose and regimen, intervention timing or season, and participant characteristics. Exploratory subgroup analyses did not identify statistically significant between-subgroup differences by baseline status or supplementation regimen, but the small number of comparisons limits inference. These findings therefore do not establish an optimal target population or supplementation strategy.

### 4.3. Athletic performance

#### 4.3.1. Jump and muscular performance.

The aggregated data did not show a statistically significant pooled effect of vitamin D supplementation on jump or muscular performance in football players. The results of this study broadly agree with those from previous meta-analyses of effects in athletes, which did not reveal any statistically significant overall effect on muscle strength or power with vitamin D supplementation [7]. However, favorable findings have been reported for some specific lower-limb outcomes or individual strength tests. For example, Zhang et al. (2019) reported a significant effect for lower-limb muscle strength but not for upper-limb strength or muscle power, and Han et al. (2024) reported a significant effect for quadriceps contraction but not for the overall combined strength outcome. In analyses restricted to maximal strength and vertical jump power, Sist et al. (2023) also reported no statistically significant effect of vitamin D supplementation.

A possible explanation for the disparity in conclusions is that, if vitamin D is indeed affecting performance, it may be context-specific and not consistently affect all neuromuscular outputs. Football performance relies on several synergistic physiological systems, and small changes in one muscular parameter can be “masked” by other parameters when measured by a wider, composite, or heterogeneous performance endpoint. In addition, many of the included studies were not specifically designed to identify small changes in the high-performance athletic population, leading to the possibility of type II error in individual outcomes and inter-study variability in the effects reported.

Because maximal strength, muscular power, quadriceps contraction, and jump performance are related but distinct outcomes, positive findings from isolated lower-limb or test-specific measures should not be assumed to apply to broader jump or muscular performance outcomes in football players. Differences in testing protocols may also limit the comparability of pooled estimates across studies. Accordingly, these results should be interpreted as indicating a lack of clear evidence of performance improvement for these outcomes, rather than as evidence that vitamin D supplementation has no effect whatsoever on muscle function.

#### 4.3.2. Sprint performance.

Vitamin D supplementation was not found to exert a statistically significant pooled effect on sprint performance in football players, and the pooled estimate was close to the null. This finding is broadly consistent with previous evidence from elite athletes, in which no between-group differences were observed in four of six sprint studies [32].

Sprint tests differed in distance and testing protocols, which may limit direct comparisons across studies. Although no statistical heterogeneity was detected in the present analysis, the analysis included only four comparisons and 113 players, which lowers the precision of the pooled estimate and reduces the likelihood of detecting heterogeneity. An I^2^ value of 0% should therefore not be interpreted as evidence that clinical or methodological heterogeneity was absent.

#### 4.3.3. Aerobic capacity.

Vitamin D supplementation did not show a statistically significant pooled effect on aerobic capacity in football players. This finding should be interpreted in the context of previous evidence. In a systematic review of elite athletes, some individual studies reported improvements in VO_2_max or other measures of aerobic capacity, but the evidence was based on a limited number of trials and included athletes from different sports [32].

Because previous reviews included mixed athletic populations, their findings may not be directly applicable to football players. In the present sport-specific analysis, the confidence interval included the null value, and confidence in the findings was extremely limited. Current data do not confirm an improvement in aerobic capacity after vitamin D supplementation in football players.

### 4.4 Biochemical and functional responses

The present findings indicate that the biochemical and functional responses to vitamin D supplementation should be interpreted separately. Although supplementation increased serum 25(OH)D concentrations, no statistically significant pooled improvements were observed in jump performance, sprint performance, aerobic capacity, or muscular performance.

Exploratory subgroup analyses were conducted only for circulating 25(OH)D and did not assess whether baseline status or supplementation regimen modified the effects on physical performance. Accordingly, these subgroup findings should not be extrapolated to functional outcomes.

### 4.5. Practical implications

Vitamin D supplementation may increase circulating 25(OH)D concentrations in players with low vitamin D status, but current evidence is insufficient to support a consistent improvement in jump performance, sprint performance, aerobic capacity, or muscular performance. Because this review did not assess bone health, injury risk, disease prevention, or other long-term health outcomes, the findings should not be extrapolated beyond the outcomes evaluated.

The present study did not directly compare supplementation doses, administration routes, intervention durations, or target serum 25(OH)D concentrations, and it did not evaluate the long-term safety of supplementation. Supplementation regimens should not be selected solely on the basis of subgroup effect estimates.

### 4.6. Strengths, limitations, and future directions

A number of design-related strengths support this review, including the use of design-specific RoB tools, sensitivity analyses, and GRADE appraisals across key outcomes.

Several concerns remain. First, only a small amount of evidence was available, comprising nine independent studies and 312 players, and the number of studies and participants available for each outcome was limited. Several performance-outcome estimates were imprecise because of the limited number of studies and participants, contributing to the low to very low certainty ratings. Second, the overwhelming majority of participants were youth or young male players, making it difficult to draw conclusions about the implications for players at other levels of competition, adult professional players, or female athletes. Third, differences in study design, supplementation protocols, participant characteristics, and performance-testing methods may have limited comparability across studies. Fourth, a direct evaluation of the relationship of serum 25(OH)D changes with physical performance change was not performed in the present analysis. Fifth, it was not possible to formally evaluate publication bias as fewer than 10 effect sizes were available for each outcome, and there may have been language bias because all full-text articles had to be in English.

Adequately powered RCTs with more diverse football populations and more standardized, football-relevant performance tests should be the focus of future research. To elucidate whether improvements in vitamin D status are linked to functional benefits, studies should prespecify baseline 25(OH)D status and supplementation exposure and assess serum 25(OH)D and performance outcomes at the same time points.

## 5. Conclusion

Vitamin D supplementation was associated with increased circulating 25(OH)D concentrations in association football and futsal players. However, current RCT evidence is insufficient to support a consistent improvement in jump performance, sprint performance, aerobic capacity, or muscular performance. Given the small evidence base and the low to very low certainty of evidence, these findings should be interpreted cautiously. Further adequately powered RCTs using clearly defined baseline vitamin D status and standardized, football-relevant performance measures are warranted.

## Supporting information

S1 File PRISMA 2020 Checklist. (DOCX)

S1 Table Database-specific search strategies. (DOCX)

S2 Table Sensitivity analyses. (DOCX)

S3 Table Leave-one-out sensitivity analysis for serum 25(OH)D concentration. (DOCX)

S4 Table Outcome-specific risk-of-bias assessments for randomized and non-randomized evidence. (DOCX)

S5 Table Study-level characteristics of comparisons included in the meta-analysis and sensitivity analyses. (DOCX)

S1 Fig Outcome-specific forest plots for serum 25(OH)D concentration and physical performance outcomes. Forest plots show standardized mean differences expressed as Hedges’ g with 95% confidence intervals for serum 25(OH)D concentration, jump performance, sprint performance, aerobic capacity, and muscular performance. Individual study-level effects, pooled estimates, study weights, heterogeneity statistics, and prediction intervals are presented.
(TIFF)
