# Supplementary material for: Effects of vitamin D supplementation on vitamin D status and physical performance in football players: A systematic review and meta-analysis
Source: PLoS One. 2026 Nov 6;21(11):e0362552. doi: 10.1371/journal.pone.0362552 (PMC13742961; doi:10.1371/journal.pone.0362552)
Supplement: Supplementary file 2 [file pone.0362552.s002.docx]

**Supplementary Table S1. Database search strategies**

| **Database** | **Main search strategy** |
| --- | --- |
| PubMed | ("Vitamin D"[Mesh] OR "Cholecalciferol"[Mesh] OR "vitamin D"[tiab] OR "vitamin D2"[tiab] OR "vitamin D3"[tiab] OR cholecalciferol[tiab] OR colecalciferol[tiab] OR ergocalciferol[tiab] OR calcidiol[tiab] OR "25-hydroxyvitamin D"[tiab] OR "25 hydroxyvitamin D"[tiab] OR "25(OH)D"[tiab]) AND ("Soccer"[Mesh] OR soccer[tiab] OR futsal[tiab] OR "association football"[tiab] OR footballer*[tiab] OR "football player"[tiab] OR "football players"[tiab] OR "football athlete"[tiab] OR "football athletes"[tiab] OR "five-a-side football"[tiab] OR "5-a-side football"[tiab] OR "five-a-side soccer"[tiab] OR "5-a-side soccer"[tiab]) |
| Web of Science | #1 TS=("vitamin D" OR "vitamin D2" OR "vitamin D3" OR cholecalciferol OR colecalciferol OR ergocalciferol OR calcidiol OR "25-hydroxyvitamin D" OR "25 hydroxyvitamin D" OR "25(OH)D") #2 TS=(soccer OR futsal OR "association football" OR footballer* OR "football player*" OR "football athlete*" OR "five-a-side football" OR "5-a-side football" OR "five-a-side soccer" OR "5-a-side soccer") #3 #1 AND #2 |
| Scopus | TITLE-ABS-KEY(("vitamin D" OR "vitamin D2" OR "vitamin D3" OR cholecalciferol OR colecalciferol OR ergocalciferol OR calcidiol OR "25-hydroxyvitamin D" OR "25 hydroxyvitamin D" OR "25(OH)D") AND (soccer OR futsal OR "association football" OR footballer* OR "football player*" OR "football athlete*" OR "five-a-side football" OR "5-a-side football" OR "five-a-side soccer" OR "5-a-side soccer")) |
| Embase | ('vitamin d'/exp OR 'cholecalciferol'/exp OR 'ergocalciferol'/exp OR 'vitamin d':ti,ab,kw OR 'vitamin d2':ti,ab,kw OR 'vitamin d3':ti,ab,kw OR cholecalciferol:ti,ab,kw OR colecalciferol:ti,ab,kw OR ergocalciferol:ti,ab,kw OR calcidiol:ti,ab,kw OR '25-hydroxyvitamin d':ti,ab,kw OR '25 hydroxyvitamin d':ti,ab,kw OR '25(oh)d':ti,ab,kw) AND ('soccer'/exp OR soccer:ti,ab,kw OR futsal:ti,ab,kw OR 'association football':ti,ab,kw OR footballer*:ti,ab,kw OR 'football player*':ti,ab,kw OR 'football athlete*':ti,ab,kw OR 'five-a-side football':ti,ab,kw OR '5-a-side football':ti,ab,kw OR 'five-a-side soccer':ti,ab,kw OR '5-a-side soccer':ti,ab,kw) |
| Cochrane Library | #1 "vitamin D" OR "vitamin D2" OR "vitamin D3" OR cholecalciferol OR colecalciferol OR ergocalciferol OR calcidiol OR "25-hydroxyvitamin D" OR "25 hydroxyvitamin D" OR "25(OH)D"  #2 soccer OR futsal OR "association football" OR footballer* OR "football player" OR "football players" OR "football athlete" OR "football athletes" OR "five-a-side football" OR "5-a-side football" OR "five-a-side soccer" OR "5-a-side soccer"  #3 #1 AND #2 |

**Note.** Search strategies were adapted to the syntax and indexing system of each database. No restrictions on sex, age, competitive level, publication year, study design, or outcome terms were applied during the database search; eligibility was determined during study screening. Searches were conducted from database inception to 31 May 2026.
