# Supplementary material for: Effects of vitamin D supplementation on vitamin D status and physical performance in football players: A systematic review and meta-analysis
Source: PLoS One. 2026 Nov 6;21(11):e0362552. doi: 10.1371/journal.pone.0362552 (PMC13742961; doi:10.1371/journal.pone.0362552)
Supplement: Supplementary file 3 [file pone.0362552.s003.docx]

**Sensitivity Analyses**

**Supplementary Table S2. Sensitivity analyses based on the revised RCT-only primary analyses.**

**Panel A. Design- and model-based sensitivity analyses**

| **Outcome** | **Analysis** | **Model** | **k** | **N** | **Hedges' g [95% CI]** | **p value** | **I²** | **Result** |
| --- | --- | --- | --- | --- | --- | --- | --- | --- |
| 25(OH)D | RCT-only primary | REML random effects | 6 | 212 | 1.41 [0.90, 1.92] | p<0.001 | 58.0% | Significant |
| 25(OH)D | RCT-only model sensitivity | Fixed effect | 6 | 212 | 1.43 [1.12, 1.73] | p<0.001 | 58.0% | Significant |
| 25(OH)D | All-study sensitivity (RCT + non-RCT/quasi) | REML random effects | 9 | 284 | 2.15 [1.31, 2.99] | p<0.001 | 87.0% | Significant |
| Jump performance | RCT-only primary | REML random effects | 4 | 154 | 0.21 [-0.10, 0.53] | p=0.186 | 0% | Not significant |
| Jump performance | RCT-only model sensitivity | Fixed effect | 4 | 154 | 0.21 [-0.10, 0.53] | p=0.186 | 0% | Not significant |
| Jump performance | All-study sensitivity (RCT + non-RCT/quasi) | REML random effects | 5 | 190 | 0.26 [-0.02, 0.55] | p=0.072 | 0% | Not significant |
| Sprint performance | RCT-only primary | REML random effects | 4 | 113 | -0.03 [-0.40, 0.34] | p=0.886 | 0% | Not significant |
| Sprint performance | RCT-only model sensitivity | Fixed effect | 4 | 113 | -0.03 [-0.40, 0.34] | p=0.886 | 0% | Not significant |
| Sprint performance | All-study sensitivity (RCT + non-RCT/quasi) | REML random effects | 5 | 149 | 0.03 [-0.29, 0.35] | p=0.852 | 0% | Not significant |
| Aerobic capacity (VO₂max) | RCT-only primary | REML random effects | 3 | 77 | 0.00 [-0.45, 0.45] | p=0.995 | 0% | Not significant |
| Aerobic capacity (VO₂max) | RCT-only model sensitivity | Fixed effect | 3 | 77 | 0.00 [-0.45, 0.45] | p=0.995 | 0% | Not significant |
| Aerobic capacity (VO₂max) | All-study sensitivity (RCT + non-RCT/quasi) | REML random effects | 6 | 149 | 0.21 [-0.12, 0.54] | p=0.211 | 2.4% | Not significant |
| Muscular performance | RCT-only primary | REML random effects | 4 | 146 | 0.19 [-0.14, 0.51] | p=0.264 | 0% | Not significant |
| Muscular performance | RCT-only model sensitivity | Fixed effect | 4 | 146 | 0.19 [-0.14, 0.51] | p=0.264 | 0% | Not significant |
| Muscular performance | All-study sensitivity (RCT + non-RCT/quasi) | REML random effects | 7 | 218 | 0.21 [-0.06, 0.48] | p=0.123 | 0% | Not significant |

**Panel B. Sensitivity to the assumed pre-post correlation used to reconstruct change-score SDs**

| **Outcome** | **Assumed r** | **k** | **N** | **Hedges' g [95% CI]** | **p value** | **I²** | **Result** |
| --- | --- | --- | --- | --- | --- | --- | --- |
| 25(OH)D | 0.25 | 6 | 212 | 1.29 [0.83, 1.75] | p<0.001 | 52.2% | Significant |
| 25(OH)D | 0.50 | 6 | 212 | 1.41 [0.90, 1.92] | p<0.001 | 58.0% | Significant |
| 25(OH)D | 0.75 | 6 | 212 | 1.70 [1.02, 2.37] | p<0.001 | 74.9% | Significant |
| Jump performance | 0.25 | 4 | 154 | 0.18 [-0.14, 0.49] | p=0.275 | 0% | Not significant |
| Jump performance | 0.50 | 4 | 154 | 0.21 [-0.10, 0.53] | p=0.186 | 0% | Not significant |
| Jump performance | 0.75 | 4 | 154 | 0.30 [-0.02, 0.61] | p=0.069 | 0% | Not significant |
| Sprint performance | 0.25 | 4 | 113 | -0.03 [-0.40, 0.34] | p=0.887 | 0% | Not significant |
| Sprint performance | 0.50 | 4 | 113 | -0.03 [-0.40, 0.34] | p=0.886 | 0% | Not significant |
| Sprint performance | 0.75 | 4 | 113 | -0.08 [-0.57, 0.42] | p=0.767 | 43.3% | Not significant |
| Aerobic capacity (VO₂max) | 0.25 | 3 | 77 | 0.00 [-0.45, 0.45] | p=0.996 | 0% | Not significant |
| Aerobic capacity (VO₂max) | 0.50 | 3 | 77 | 0.00 [-0.45, 0.45] | p=0.995 | 0% | Not significant |
| Aerobic capacity (VO₂max) | 0.75 | 3 | 77 | 0.00 [-0.45, 0.45] | p=0.991 | 0% | Not significant |
| Muscular performance | 0.25 | 4 | 146 | 0.15 [-0.17, 0.48] | p=0.363 | 0% | Not significant |
| Muscular performance | 0.50 | 4 | 146 | 0.19 [-0.14, 0.51] | p=0.264 | 0% | Not significant |
| Muscular performance | 0.75 | 4 | 146 | 0.26 [-0.06, 0.59] | p=0.114 | 0% | Not significant |

**Panel C. Absolute-scale analysis of serum 25(OH)D concentration**

| **Outcome** | **Analysis** | **Effect measure** | **k** | **N** | **Estimate [95% CI]** | **p value** | **I²** |
| --- | --- | --- | --- | --- | --- | --- | --- |
| 25(OH)D | RCT-only absolute-scale sensitivity | Mean difference (ng/mL) | 6 | 212 | 12.14 [5.84, 18.43] | p<0.001 | 89.8% |

**Notes.** Primary analyses were restricted to randomized comparisons and used random-effects models with REML. The all-study sensitivity analysis reintroduced eligible non-randomized/quasi-experimental comparisons. For correlation sensitivity analyses, only change-score SDs that required reconstruction were recalculated at r = 0.25, 0.50, and 0.75; directly reported change-score SDs were retained. The r = 0.50 rows correspond to the primary assumption. The absolute 25(OH)D mean-difference analysis used ng/mL after unit harmonization. k = number of comparisons; N = total participants contributing to the pooled estimate; CI = confidence interval. The Jastrzębska 2016/2018/Skalska 2019 reports were treated as one overlapping non-randomized trial, and the two Motavari comparisons used disjoint arms without a shared control. All-study sensitivity estimates were not used for GRADE certainty ratings.
