# Supplementary material for: Effects of vitamin D supplementation on vitamin D status and physical performance in football players: A systematic review and meta-analysis
Source: PLoS One. 2026 Nov 6;21(11):e0362552. doi: 10.1371/journal.pone.0362552 (PMC13742961; doi:10.1371/journal.pone.0362552)
Supplement: Supplementary file 4 [file pone.0362552.s004.docx]

**Supplementary Table S3. Leave-one-out sensitivity analysis for the RCT-only 25(OH)D meta-analysis.**

| **Omitted comparison** | **k** | **N** | **Hedges' g** | **95% CI** | **p value** | **I²** |
| --- | --- | --- | --- | --- | --- | --- |
| Alimoradi et al., 2019 | 5 | 143 | 1.39 | 0.74 to 2.04 | p<0.001 | 64.2% |
| Bezrati et al., 2020 | 5 | 176 | 1.31 | 0.75 to 1.88 | p<0.001 | 60.4% |
| Bezuglov et al., 2025 | 5 | 164 | 1.36 | 0.74 to 1.98 | p<0.001 | 64.4% |
| Brzeziański et al., 2022 | 5 | 187 | 1.40 | 0.79 to 2.01 | p<0.001 | 67.6% |
| Close et al., 2013 | 5 | 202 | 1.37 | 0.82 to 1.93 | p<0.001 | 66.0% |
| Jastrzębska et al., 2022 | 5 | 188 | 1.63 | 1.30 to 1.96 | p<0.001 | 0% |

**Note.** Each row omits one randomized comparison from the six-comparison RCT-only 25(OH)D primary meta-analysis. All leave-one-out estimates remained statistically significant.
