# Supplementary material for: Effects of vitamin D supplementation on vitamin D status and physical performance in football players: A systematic review and meta-analysis
Source: PLoS One. 2026 Nov 6;21(11):e0362552. doi: 10.1371/journal.pone.0362552 (PMC13742961; doi:10.1371/journal.pone.0362552)
Supplement: Supplementary file 5 [file pone.0362552.s005.docx]

**Supplementary Table S4. Outcome-specific risk-of-bias assessments for randomized and non-randomized evidence**

**Panel A. Outcome-specific RoB 2 judgments for RCT results in the primary analyses**

| **Study** | **Outcome/result** | **D1** | **D2** | **D3** | **D4** | **D5** | **Overall** |
| --- | --- | --- | --- | --- | --- | --- | --- |
| Alimoradi et al., 2019 | 25(OH)D concentration | Some concerns | Low | Low | Low | Some concerns | **Some concerns** |
| Alimoradi et al., 2019 | Jump performance | Some concerns | Low | Low | Low | Some concerns | **Some concerns** |
| Alimoradi et al., 2019 | Muscular performance | Some concerns | Low | Low | Low | Some concerns | **Some concerns** |
| Bezrati et al., 2020 | 25(OH)D concentration | Some concerns | Low | Some concerns | Low | Some concerns | **Some concerns** |
| Bezrati et al., 2020 | Jump performance | Some concerns | Low | Some concerns | Low | Some concerns | **Some concerns** |
| Bezrati et al., 2020 | Sprint performance | Some concerns | Low | Some concerns | Low | Some concerns | **Some concerns** |
| Bezuglov et al., 2025 | 25(OH)D concentration | Some concerns | Some concerns | Low | Low | Some concerns | **Some concerns** |
| Brzeziański et al., 2022 | 25(OH)D concentration | Some concerns | Low | Low | Low | Some concerns | **Some concerns** |
| Brzeziański et al., 2022 | Jump performance | Some concerns | Low | Low | Low | Some concerns | **Some concerns** |
| Brzeziański et al., 2022 | Sprint performance | Some concerns | Low | Low | Low | Some concerns | **Some concerns** |
| Brzeziański et al., 2022 | Aerobic capacity (VO₂max) | Some concerns | Low | Low | Low | Some concerns | **Some concerns** |
| Brzeziański et al., 2022 | Muscular performance | Some concerns | Low | Low | Low | Some concerns | **Some concerns** |
| Close et al., 2013 | 25(OH)D concentration | Some concerns | Low | Low | Low | Some concerns | **Some concerns** |
| Jastrzębska et al., 2022 | 25(OH)D concentration | Some concerns | Some concerns | High | Low | Some concerns | **High** |
| Jastrzębska et al., 2022 | Jump performance | Some concerns | Some concerns | High | Low | Some concerns | **High** |
| Jastrzębska et al., 2022 | Sprint performance | Some concerns | Some concerns | High | Low | Some concerns | **High** |
| Jastrzębska et al., 2022 | Aerobic capacity (VO₂max) | Some concerns | Some concerns | High | Low | Some concerns | **High** |
| Jastrzębska et al., 2022 | Muscular performance | Some concerns | Some concerns | High | Low | Some concerns | **High** |
| Michalczyk et al., 2020 | Sprint performance | Some concerns | Some concerns | Low | Low | Some concerns | **Some concerns** |
| Michalczyk et al., 2020 | Aerobic capacity (VO₂max) | Some concerns | Some concerns | Low | Low | Some concerns | **Some concerns** |
| Michalczyk et al., 2020 | Muscular performance | Some concerns | Some concerns | Low | Low | Some concerns | **Some concerns** |

**Panel B. Outcome-specific ROBINS-I judgments for non-RCT/quasi-experimental results in sensitivity analyses**

| **Study** | **Outcome/result** | **D1** | **D2** | **D3** | **D4** | **D5** | **D6** | **D7** | **Overall** |
| --- | --- | --- | --- | --- | --- | --- | --- | --- | --- |
| Jastrzębska et al., 2016 / Jastrzębska et al., 2018 / Skalska et al., 2019 (same trial) | 25(OH)D concentration | Moderate | Moderate | Low | Low | Moderate | Low | Moderate | **Moderate** |
| Jastrzębska et al., 2016 / Jastrzębska et al., 2018 / Skalska et al., 2019 (same trial) | Jump performance | Moderate | Moderate | Low | Low | Moderate | Low | Moderate | **Moderate** |
| Jastrzębska et al., 2016 / Jastrzębska et al., 2018 / Skalska et al., 2019 (same trial) | Sprint performance | Moderate | Moderate | Low | Low | Moderate | Low | Moderate | **Moderate** |
| Jastrzębska et al., 2016 / Jastrzębska et al., 2018 / Skalska et al., 2019 (same trial) | Aerobic capacity (VO₂max) | Moderate | Moderate | Low | Low | Moderate | Low | Moderate | **Moderate** |
| Jastrzębska et al., 2016 / Jastrzębska et al., 2018 / Skalska et al., 2019 (same trial) | Muscular performance | Moderate | Moderate | Low | Low | Moderate | Low | Moderate | **Moderate** |
| Motavari et al., 2022 | 25(OH)D concentration | Moderate | Moderate | Low | Moderate | Low | Low | Moderate | **Moderate** |
| Motavari et al., 2022 | Aerobic capacity (VO₂max) | Moderate | Moderate | Low | Moderate | Low | Low | Moderate | **Moderate** |
| Motavari et al., 2022 | Muscular performance | Moderate | Moderate | Low | Moderate | Low | Low | Moderate | **Moderate** |

**Notes.** RoB 2 domains: D1, bias arising from the randomization process; D2, bias due to deviations from intended interventions; D3, bias due to missing outcome data; D4, bias in measurement of the outcome; D5, bias in selection of the reported result. ROBINS-I domains: D1, bias due to confounding; D2, bias in selection of participants into the study; D3, bias in classification of interventions; D4, bias due to deviations from intended interventions; D5, bias due to missing data; D6, bias in measurement of outcomes; D7, bias in selection of the reported result. Only results contributing to the revised quantitative syntheses are shown. The Jastrzębska 2016/2018/Skalska 2019 reports were treated as one overlapping non-randomized trial.
