# Supplementary material for: Effects of vitamin D supplementation on vitamin D status and physical performance in football players: A systematic review and meta-analysis
Source: PLoS One. 2026 Nov 6;21(11):e0362552. doi: 10.1371/journal.pone.0362552 (PMC13742961; doi:10.1371/journal.pone.0362552)
Supplement: Supplementary file 6 [file pone.0362552.s006.docx]

**Supplementary Table S5. Study-level characteristics of comparisons included in the meta-analysis and sensitivity analyses**

Panel A. 25(OH)D concentration

| **Study** | **Design / analysis set** | **Comparison** | **N (E/C)** | **Total N** | **Outcome measure** | **Unit** | **Hedges' g [95% CI]** |
| --- | --- | --- | --- | --- | --- | --- | --- |
| Alimoradi et al., 2019 | RCT / Primary | VitD vs placebo | 35/34 | 69 | 25(OH)D | ng/mL | 1.50 [0.97, 2.04] |
| Bezrati et al., 2020 | RCT / Primary | VitD vs placebo | 19/17 | 36 | 25(OH)D | ng/mL | 1.92 [1.11, 2.72] |
| Bezuglov et al., 2025 | RCT / Primary | VitD vs no supplementation | 25/23 | 48 | 25(OH)D | ng/mL | 1.65 [0.99, 2.31] |
| Brzeziański et al., 2022 | RCT / Primary | VitD vs placebo/no vitamin D | 12/13 | 25 | 25(OH)D | ng/mL | 1.53 [0.62, 2.44] |
| Close et al., 2013 | RCT / Primary | VitD vs placebo | 5/5 | 10 | 25(OH)D | ng/mL | 1.85 [0.23, 3.46] |
| Jastrzębska et al., 2022 | RCT / Primary | VitD vs placebo | 11/13 | 24 | 25(OH)D | ng/mL | 0.20 [-0.60, 1.01] |
| Jastrzębska 2016 / Jastrzębska 2018 / Skalska 2019 (same trial) | Non-RCT / Sensitivity | VitD vs placebo | 20/16 | 36 | 25(OH)D | ng/mL | 3.30 [2.26, 4.35] |
| Motavari et al., 2022 (SUP vs CON) | Quasi / Sensitivity | SUP vs CON | 9/9 | 18 | 25(OH)D | ng/mL | 4.70 [2.73, 6.66] |
| Motavari et al., 2022 (EXS vs EX) | Quasi / Sensitivity | EXS vs EX | 9/9 | 18 | 25(OH)D | ng/mL | 3.93 [2.21, 5.65] |

Panel B. Jump performance

| **Study** | **Design / analysis set** | **Comparison** | **N (E/C)** | **Total N** | **Outcome measure** | **Unit** | **Hedges' g [95% CI]** |
| --- | --- | --- | --- | --- | --- | --- | --- |
| Alimoradi et al., 2019 | RCT / Primary | VitD vs placebo | 35/34 | 69 | Vertical jump | % | 0.30 [-0.17, 0.77] |
| Bezrati et al., 2020 | RCT / Primary | VitD vs placebo | 19/17 | 36 | Vertical jump | cm | 0.05 [-0.61, 0.70] |
| Brzeziański et al., 2022 | RCT / Primary | VitD vs placebo/no vitamin D | 12/13 | 25 | CMJ | cm | 0.37 [-0.42, 1.16] |
| Jastrzębska et al., 2022 | RCT / Primary | VitD vs placebo | 11/13 | 24 | CMJ | cm | 0.06 [-0.74, 0.87] |
| Jastrzębska 2016 / Jastrzębska 2018 / Skalska 2019 (same trial) | Non-RCT / Sensitivity | VitD vs placebo | 20/16 | 36 | CMJ | cm | 0.48 [-0.19, 1.14] |

Panel C. Sprint performance

| **Study** | **Design / analysis set** | **Comparison** | **N (E/C)** | **Total N** | **Outcome measure** | **Unit** | **Hedges' g [95% CI]** |
| --- | --- | --- | --- | --- | --- | --- | --- |
| Bezrati et al., 2020 | RCT / Primary | VitD vs placebo | 19/17 | 36 | 10 m sprint | s | 0.36 [-0.30, 1.02] |
| Brzeziański et al., 2022 | RCT / Primary | VitD vs placebo/no vitamin D | 12/13 | 25 | 10 m sprint | s | -0.44 [-1.24, 0.35] |
| Jastrzębska et al., 2022 | RCT / Primary | VitD vs placebo (T2→T3) | 11/13 | 24 | 10 m sprint | s | -0.21 [-1.02, 0.59] |
| Michalczyk et al., 2020 | RCT / Primary | VitD vs placebo | 15/13 | 28 | 30 m sprint | s | 0.00 [-0.74, 0.74] |
| Jastrzębska 2016 / Jastrzębska 2018 / Skalska 2019 (same trial) | Non-RCT / Sensitivity | VitD vs placebo | 20/16 | 36 | 10 m sprint | s | 0.21 [-0.45, 0.87] |

Panel D. Aerobic capacity (VO₂max)

| **Study** | **Design / analysis set** | **Comparison** | **N (E/C)** | **Total N** | **Outcome measure** | **Unit** | **Hedges' g [95% CI]** |
| --- | --- | --- | --- | --- | --- | --- | --- |
| Brzeziański et al., 2022 | RCT / Primary | VitD vs placebo/no vitamin D | 12/13 | 25 | VO₂max | mL/kg/min | 0.06 [-0.72, 0.85] |
| Jastrzębska et al., 2022 | RCT / Primary | VitD vs placebo | 11/13 | 24 | VO₂max | mL/kg/min | -0.09 [-0.89, 0.71] |
| Michalczyk et al., 2020 | RCT / Primary | VitD vs placebo | 15/13 | 28 | VO₂max | mL/kg/min | 0.03 [-0.72, 0.77] |
| Jastrzębska 2016 / Jastrzębska 2018 / Skalska 2019 (same trial) | Non-RCT / Sensitivity | VitD vs placebo | 20/16 | 36 | VO₂max | mL/kg/min | 0.80 [0.11, 1.49] |
| Motavari et al., 2022 (SUP vs CON) | Quasi / Sensitivity | SUP vs CON | 9/9 | 18 | VO₂max | mL/kg/min | 0.31 [-0.62, 1.25] |
| Motavari et al., 2022 (EXS vs EX) | Quasi / Sensitivity | EXS vs EX | 9/9 | 18 | VO₂max | mL/kg/min | -0.07 [-1.00, 0.85] |

Panel E. Muscular performance

| **Study** | **Design / analysis set** | **Comparison** | **N (E/C)** | **Total N** | **Outcome measure** | **Unit** | **Hedges' g [95% CI]** |
| --- | --- | --- | --- | --- | --- | --- | --- |
| Alimoradi et al., 2019 | RCT / Primary | VitD vs placebo | 35/34 | 69 | Leg-press strength | % | 0.31 [-0.17, 0.78] |
| Brzeziański et al., 2022 | RCT / Primary | VitD vs placebo/no vitamin D | 12/13 | 25 | 10-jump power | W/kg | 0.20 [-0.59, 0.99] |
| Jastrzębska et al., 2022 | RCT / Primary | VitD vs placebo | 11/13 | 24 | 10 jumps | cm | 0.26 [-0.55, 1.07] |
| Michalczyk et al., 2020 | RCT / Primary | VitD vs placebo | 15/13 | 28 | PLL | W | -0.19 [-0.93, 0.56] |
| Jastrzębska 2016 / Jastrzębska 2018 / Skalska 2019 (same trial) | Non-RCT / Sensitivity | VitD vs placebo | 20/16 | 36 | Wingate peak power | W/kg | 0.34 [-0.33, 1.00] |
| Motavari et al., 2022 (SUP vs CON) | Quasi / Sensitivity | SUP vs CON | 9/9 | 18 | Lower-body muscle strength | kg | 0.35 [-0.58, 1.28] |
| Motavari et al., 2022 (EXS vs EX) | Quasi / Sensitivity | EXS vs EX | 9/9 | 18 | Lower-body muscle strength | kg | 0.03 [-0.90, 0.95] |

**Notes.** RCT = randomized controlled trial; E/C = experimental/control; CMJ = countermovement jump; VO₂max = maximal oxygen uptake; PLL = power of the left leg; SUP = supplementation; CON = control; EXS = exercise plus supplementation; EX = exercise. Randomized comparisons contributed to the primary analyses; the overlapping Jastrzębska 2016/Jastrzębska 2018/Skalska 2019 trial and Motavari 2022 contributed only to all-study sensitivity analyses. The two Motavari comparisons used disjoint arms and did not share a control group. Hedges' g values are standardized mean differences with 95% confidence intervals; positive values favor vitamin D supplementation.
